# Ethical Dilemmas in Covert Prescribing: A Case Report on Medication Administration for Noncompliant Psychiatric Patients

**DOI:** 10.7759/cureus.70905

**Published:** 2024-10-05

**Authors:** Aymun M Razzak, Meghan Mallya, Omar Abdelaziz, Michael Miller

**Affiliations:** 1 Department of Psychiatry, University of Texas Medical Branch at Galveston, Galveston, USA

**Keywords:** covert prescribing, non-consensual treatment, patient autonomy, psychiatric ethics, schizophrenia

## Abstract

Psychiatric care often presents ethical challenges, particularly when patients with severe mental illness refuse essential treatment. This case report examines the ethical complexities surrounding covert prescribing in a noncompliant psychiatric patient. A 54-year-old male with schizophrenia, admitted following an auto-pedestrian accident resulting in multiple fractures, illustrates these dilemmas. His refusal of medication despite experiencing severe psychosis led healthcare providers to administer antipsychotics covertly, with the consent of his medical power of attorney. The report explores the ethical implications of such practices, especially concerning patient autonomy and safety. It underscores the urgent need for interdisciplinary collaboration and the establishment of standardized guidelines to ethically navigate covert prescribing in psychiatric patients.

## Introduction

Covert prescribing, defined as the administration of medications without the patient’s knowledge, has long been a subject of ethical debate, particularly in populations with cognitive impairments such as dementia [1]. In these cases, covert prescribing is often justified by the principle of beneficence, which prioritizes the patient’s best interests to prevent harm when they are unable to make informed decisions about their care. However, the ethical complexities deepen in psychiatric populations, especially among patients with partial decision-making capacity who refuse treatment due to their illness [2]. This scenario creates critical ethical tensions between autonomy, beneficence, and non-maleficence.

Patient autonomy, a fundamental principle in medical ethics, emphasizes an individual’s right to refuse treatment, even when such decisions may jeopardize their health. Conversely, the principle of beneficence requires clinicians to act in ways that benefit the patient, which may necessitate interventions that override a patient’s refusal to avert greater harm [2]. In psychiatric patients, such as those with schizophrenia, the challenge lies in determining when covert prescribing might be ethically permissible, given the patient’s impaired judgment due to delusions or paranoia [3].

Existing literature predominantly addresses covert medication in populations with severe cognitive impairment, such as individuals with dementia, where the ethical rationale is more clearly defined due to the patient’s inability to make informed decisions [4]. Studies in dementia care suggest that covert prescribing can be ethically justified when it is the only viable option to ensure treatment adherence and patient safety [5]. However, fewer studies delve into the ethical nuances of covert prescribing for psychiatric patients, particularly those with schizophrenia who may retain some degree of decision-making capacity [6]. This gap underscores the need for a more robust ethical framework in psychiatric care, where patient autonomy and the duty to provide beneficial treatment often conflict [7]. This case report explores these ethical dimensions through the example of a 54-year-old male with schizophrenia, illustrating the complexities of covert prescribing in psychiatric care and the urgent need for clearer guidelines.

## Case presentation

A 54-year-old male with a long-standing history of schizophrenia was admitted following a vehicular accident that resulted in multiple fractures. Upon admission, he displayed acute psychosis characterized by disorganized thinking, religious delusions, preoccupation with feces, and an unkempt appearance. He consistently refused all medications from both the trauma and psychiatry teams, believing that he would be “healed through God.” The patient was also noted to have a metallic cross in his distal esophagus, which required endoscopic removal. He consented to the removal of the metallic cross, and an endoscopy was performed without complications. However, he continued to refuse all other treatments. Despite evident psychosis, he was not medically stable for transfer to a psychiatric facility until cleared by the trauma surgery team.

After a multidisciplinary discussion, the patient agreed to the casting of his fractures once the casts were “blessed” by a pastor. However, he refused all oral medications, considering them “ungodly.” During his admission, he began eating pages of the bible and smeared feces on the walls of his bedroom. Due to the patient’s religious delusions leading to the consumption of additional foreign bodies, safety concerns were raised. The psychiatry team recommended starting risperidone or ziprasidone to address his symptoms, with plans to consider involuntary hospitalization once he was medically stabilized. Due to his continued refusal, the trauma team sought and obtained permission from his medical power of attorney, his mother, to initiate covert administration of antipsychotics by incorporating them into his food. Risperidone was covertly started at a dose of 1 mg BID and titrated to 3 mg BID. Although the religious delusions persisted, the consumption of foreign objects ceased, suggesting some improvement.

While this approach resulted in mild symptom improvement, the patient began to experience extrapyramidal symptoms (EPS), including mild tremors in the bilateral upper extremities. Cogentin was discussed as a potential intervention for the EPS but was not initiated, as the patient refused this medication. His psychotic symptoms persisted, and he continued to resist additional treatments for his injuries, including pain management and fracture stabilization. Eventually, the patient was medically cleared by the trauma surgery team and was transferred to a state hospital for continued psychiatric care.

## Discussion

The covert administration of medications without patient consent raises significant ethical concerns, including violations of autonomy and informed consent. In this case, covert prescribing aimed to improve the patient’s psychiatric symptoms and ensure his safety, with the involvement of his medical power of attorney providing legal justification for the administration of antipsychotics. However, the patient’s continued symptoms, despite medication adjustments, raised concerns about the efficacy of this practice and its long-term ethical implications.

A PubMed search conducted in March 2024 identified 66 articles relevant to covert prescribing practices. Existing literature predominantly focuses on cases involving patients with dementia or severe cognitive impairments, with considerably less attention given to psychiatric patients, particularly those with partial decision-making capacity [8-11]. Covert medication, where drugs are administered without a patient’s knowledge, presents substantial ethical dilemmas, especially when patients refuse medication due to religious delusions. Ethical frameworks often lack concrete guidance for such cases, leaving healthcare providers to navigate the delicate balance between respecting patient autonomy and ensuring beneficence [12,13].

The legal framework surrounding covert prescribing typically provides guidance based on the patient's best interests; however, these legal protections can conflict with the ethical obligation to respect autonomy, especially in patients who retain partial decision-making capacity [14]. Cultural considerations, safety concerns, and local laws may also influence medication administration approaches. In the United States, statutes regarding the administration of psychoactive medications vary by state. In Texas, for instance, patients must consent to the administration of psychoactive medications unless there is a medication-related emergency, the patient is a minor, or consent is provided by the patient’s representative [15]. In this case, while covert medication led to temporary stabilization, the patient’s ongoing refusal of other treatments and the persistence of psychotic symptoms suggest that covert prescribing may not have been fully effective. This raises questions about the long-term ethical justification for the practice, particularly when it fails to yield sustained improvement.

A study conducted in Norwegian nursing homes emphasizes the use of covert medication, particularly in populations unable to make informed decisions, and questions whether such practices can ever be ethically justified [12]. Discussions regarding psychiatric patients, as highlighted in a study by Haw and Stubbs, indicate that covert medication is generally regarded as a last resort in severe mental illness, but only when the refusal of treatment poses a serious risk of harm [13]. Consequently, the legal boundaries surrounding treatment administration without explicit consent are guided by the necessity to act in the patient’s best interests, yet these guidelines frequently conflict with respect for autonomy, particularly in patients who retain partial decision-making capacity [16]. There is an urgent need for a robust ethical framework that promotes interdisciplinary collaboration among psychiatrists, ethicists, and legal experts. The reality is that covert prescribing can occur in various settings, with the potential for misuse or abuse that may lead to patient harm. We advocate for this practice to be acknowledged and studied further. By fostering collaboration across disciplines and involving patients or individuals with lived experiences, we can develop clearer, context-specific guidelines for covert prescribing in psychiatric care, where the principles of respecting patient autonomy and ensuring beneficence often conflict [17].

The literature also reveals disagreement regarding the appropriateness of long-term covert medication administration and whether patients should be informed of covert practices if or when they regain insight [18]. These topics should be integrated into a framework or guidance on covert prescribing. While covert prescribing should never be the first resort, it may be indicated in challenging cases. Therefore, this practice must be carefully documented to track treatment responses and continually reevaluated, as decisional capacity is fluid and can change with varying conditions [19]. Guidelines must also address the unique challenges of treating psychiatric patients with partial capacity, providing clinicians with ethical clarity in situations where covert prescribing might be warranted.

Written informed consent was obtained from the patient’s mother, who also served as his medical power of attorney.

## Conclusions

This case highlights the ethical and clinical challenges associated with covert prescribing in non-committed psychiatric patients. The need to balance patient autonomy with the obligation to provide essential medical treatment presents a significant ethical dilemma. This report advocates for the development of standardized protocols to guide healthcare providers in navigating these complex decisions, ensuring respect for patient rights while prioritizing their well-being.
